# Nerve growth factor from Chinese cobra venom stimulates chondrogenic differentiation of mesenchymal stem cells

**DOI:** 10.1038/cddis.2017.208

**Published:** 2017-05-18

**Authors:** Zhenhui Lu, Danqing Lei, Tongmeng Jiang, Lihui Yang, Li Zheng, Jinmin Zhao

**Affiliations:** 1 Guangxi Engineering Center in Biomedical Material for Tissue and Organ Regeneration, The First Affiliated Hospital of Guangxi Medical University, Nanning, China; 2 Guangxi Collaborative Innovation Center for Biomedicine, The First Affiliated Hospital of Guangxi Medical University, Nanning, China; 3 Guangxi Key Laboratory of Regenerative Medicine, The First Affiliated Hospital of Guangxi Medical University, Nanning, China; 4 The Medical and Scientific Research Center, Guangxi Medical University, Nanning, China; 5 Department of Orthopaedics Trauma and Hand Surgery, The First Affiliated Hospital of Guangxi Medical University, Nanning, China; 6 School of Nursing, Guangxi Medical University, Nanning, China

## Abstract

Growth factors such as transforming growth factor beta1 (TGF-*β*1), have critical roles in the regulation of the chondrogenic differentiation of mesenchymal stem cells (MSCs), which promote cartilage repair. However, the clinical applications of the traditional growth factors are limited by their high cost, functional heterogeneity and unpredictable effects, such as cyst formation. It may be advantageous for cartilage regeneration to identify a low-cost substitute with greater chondral specificity and easy accessibility. As a neuropeptide, nerve growth factor (NGF) was involved in cartilage metabolism and NGF is hypothesized to mediate the chondrogenic differentiation of MSCs. We isolated NGF from Chinese cobra venom using a three-step procedure that we had improved upon from previous studies, and investigated the chondrogenic potential of NGF on bone marrow MSCs (BMSCs) both *in vitro* and *in vivo*. The results showed that NGF greatly upregulated the expression of cartilage-specific markers. When applied to cartilage repair for 4, 8 and 12 weeks, NGF-treated BMSCs have greater therapeutic effect than untreated BMSCs. Although inferior to TGF-*β*1 regarding its chondrogenic potential, NGF showed considerably lower expression of collagen type I, which is a fibrocartilage marker, and *RUNX2*, which is critical for terminal chondrocyte differentiation than TGF-*β*1, indicating its chondral specificity. Interestingly, NGF rarely induced BMSCs to differentiate into a neuronal phenotype, which may be due to the presence of other chondrogenic supplements. Furthermore, the underlying mechanism revealed that NGF-mediated chondrogenesis may be associated with the activation of PI3K/AKT and MAPK/ERK signaling pathways via the specific receptor of NGF, TrkA. In addition, NGF is easily accessed because of the abundance and low price of cobra venom, as well as the simplified methods for separation and purification. This study was the first to demonstrate the chondrogenic potential of NGF, which may provide a reference for cartilage regeneration in the clinic.

Adult human mesenchymal stem cells (MSCs) attracted the most attention for cartilage tissue engineering studies, because of their high proliferation rate, easy availability and capacity to differentiate into multiple cell types.^1^ For MSC-based therapy, the strategies involve the use of growth factors and 3D scaffold systems. Growth factors have critical roles of inducers that regulate the chondrogenic differentiation of MSCs. However, traditional growth factors such as TGF-*β*1 fall short in meeting the needs of clinical applications because they are limited by their high cost, rapid degradation and ready loss of activity. Moreover, the versatility and functional heterogeneity of growth factors may lead to osteophyte formation instead of chondrogenesis during cartilage regeneration.^2, 3, 4, 5, 6^ Therefore, low-cost growth factors with more specific effects on chondrogenesis may be advantageous.

Cartilage metabolism is controlled by many locally acting cytokines and growth factors, which may derive from the surrounding nerve terminals. The crucial effects of sensory and sympathetic neurotransmitters on proper limb formation during embryonic skeletal growth have been well documented.^7^ This is also confirmed by the detection of neuropeptide containing nerve fibers in the interior of the cartilage and periosteum.^8^ Clinical observations suggest that nerve fibers are important for the regulation of skeletal metabolism.^9^ Patients with neurological disorders exhibit skeletal pathophysiology.^7, 10^ Nel-like molecule-1 (Nell-1), a growth factor that is strongly expressed in neural tissue, was shown to promote chondrocyte proliferation, ECM deposition^11, 12^ and regulate chondrogenic differentiation.^13^ Nerve growth factor (NGF) is a peptide neurotrophin (NT) that could accelerate the wound healing process,^14^ regulate the growth of cells from tissues other than nerves.^15, 16, 17, 18^ The influences of neurotrophic factors on adult mammalian spinal cords were also studied.^19^ However, NGF was rarely studied in cartilage regeneration, and the effect of NGF on chondrogenesis has not been investigated.

Emerging evidences showed that NGF, either alone or in combination with BMP and NCP, shows an effect on the quantity of cartilage developed.^20^ NGF and its two receptors, tropomyosin kinase receptor A (TrkA) and p75 pan-NT receptor (p75^NTR^), were expressed in chondrocytes or chondrocyte-like cells in hyaline, fibrous and elastic cartilaginous tissues.^21, 22, 23^ In most cases, NGF exerts its biological action by triggering its specific receptor, TrkA, which activated the major cytosolic/endosomal pathways, including MAPK, ERK and PI3K/AKT.^24, 25, 26^ These signaling pathways are also involved in chondrogenic differentiation.^27, 28^ Based on these findings, the neuropeptide NGF is likely to have major implications for chondrogenic differentiation and cartilage regeneration.

Another advantage of NGF is its easy accessibility. Unlike TGF-*β*1, which has limited sources, NGF exists in many animals and can be obtained from the submaxillary salivary gland of male mice and snake venom.^29^ Most NGFs have been isolated and characterized from snake venoms that are considered to be a rich source of NGF.^30^ Venoms from the Chinese cobra (*Naja atra*) is abundant in southern China, which potentially lower the cost.^31, 32, 33^ Previously, we isolated NGF from Chinese cobra venom by gel filtration and ion exchange chromatography.^33, 34^ The simplified two-step method is useful, but the extracts were not pure and were contaminated with other proteins. A more effective method for obtaining a large amount of purified NGF that can be easily industrialized should be developed.

In this study, we extracted NGF from Chinese cobra venom by simplified three-step chromatography improved upon our previous studies. Further, the potential effects of NGF on the chondrogenic differentiation of bone marrow MSCs (BMSCs) and cartilage regeneration were investigated *in vitro* and *in vivo*. The underlying mechanism was also explored. Our findings suggested that NGF affects chondrogenesis and cartilage reconstitution, providing reference for clinical application.

## Results

### Preparation of NGF

**Figure 1:**
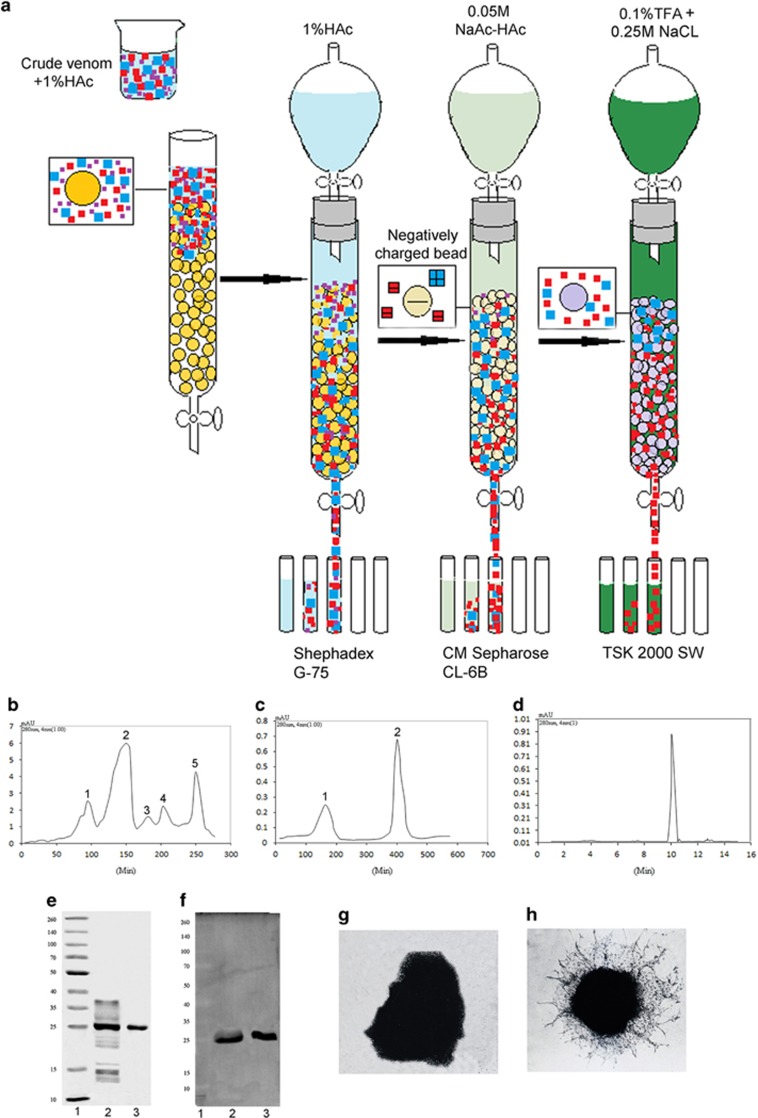
The purification and identification of NGF from cobra venom. (**a**) The schemes of NGF separation from crude Chinese cobra venom. (**b**) Chromatogram of crude venom on a Sephadex G-75 column. Sample: 2.0 g of crude Chinese cobra venom. Column: 150 cm × 3.5 cm i.d. Elution buffer: 1% HAc. Flow-rate: 2 ml/min. The left vertical axes show: UV adsorption at 280 nm (AU). Symbols 1, 2, 3, 4 and 5 separately denote the isolated chromatographic peaks. (**c**) Chromatogram of the NGF fraction on a CM Sepharose CL-6B column. Sample: 30 ml of peak 5 collected from the Sephadex G-75 column. Column: 20 cm × 1.0 cm i.d. Buffer: 0.05 M NaAc-HAc (pH 5.0). Flow-rate: 1.5 ml/min. The left vertical axes show: UV adsorption at 280 nm (AU). Symbols 1 and 2 separately denote the isolated chromatographic peaks. (**d**) Chromatogram of the TSK-G2000-SW column. Sample: 50 *μ*l of peak 2 collected from the CM Sepharose CL-6B column. Column: 600 mm × 7.5 mm i.d. Buffer: 0.1% TFA-0.25 M NaCl. Flow-rate: 1 ml/min. The left vertical axes show: UV adsorption at 280 nm (AU). (**e**) SDS-page of the fractions collected from the CM Sepharose CL-6B and TSK-G2000-SW columns. Lane 1: molecular weight marker. Lane 2: fraction containing peak 2 in **c**. Lane 3: fraction from the TSK-G2000-SW column. (**f**) Western blots was used to identify NGF and further determine the molecular weight of NGF. Lane 1: molecular weight marker. Lane 2: crude venom. Lane 3: the fractions collected from the TSK-G2000-SW column. (**g** and **h**) Effect of NGF on 8-day-old chick dorsal root ganglia. Microphotographs of the ganglia treated with the optimum concentration of NGF, 10 ng/ml (**g**) and 0 ng/ml NGF (**h**)

The procedure for purifying NGF from Chinese cobra venom was shown in Figure 1a. At each purification step, only the fraction with NGF bioactivity was collected. As shown in the Sephadex G-75 size exclusion chromatogram (Figure 1b), NGF was eluted in the fraction containing peak 5 and was accompanied with other components. The next step is ion exchange on CM Sepharose CL-6B, as shown in Figure 1c. The fraction containing peak 2, which displayed NGF bioactivity was collected, which was analyzed with high-performance liquid chromatography (HPLC) on a TSK-G2000-SW to purify and test the purity of NGF (Figure 1d). The purity was approximately 99%. On sodium dodecyl sulfate polyacrylamide gel electrophoresis (SDS-PAGE), the molecular weight of pure NGF is approximately 25 kD (Figure 1e). The yield was high, resulting in approximately 12 mg of NGF from 2.0 g of venom.

To verify the protein, western blot analysis with a mouse anti-viper antibody against NGF was performed. In Figure 1f, the band at 25 kD was confirmed as NGF. The bioactivity of NGF was tested using neurite outgrowth of chicken dorsal root ganglia (Figures 1g and h). Neurite length was significantly increased in the NGF-supplemented medium, accompanied by an increased axonal arbor density.

### The effects of NGF on monolayer cultures of BMSCs *in vitro*

#### Cell cytotoxicity

**Figure 2:**
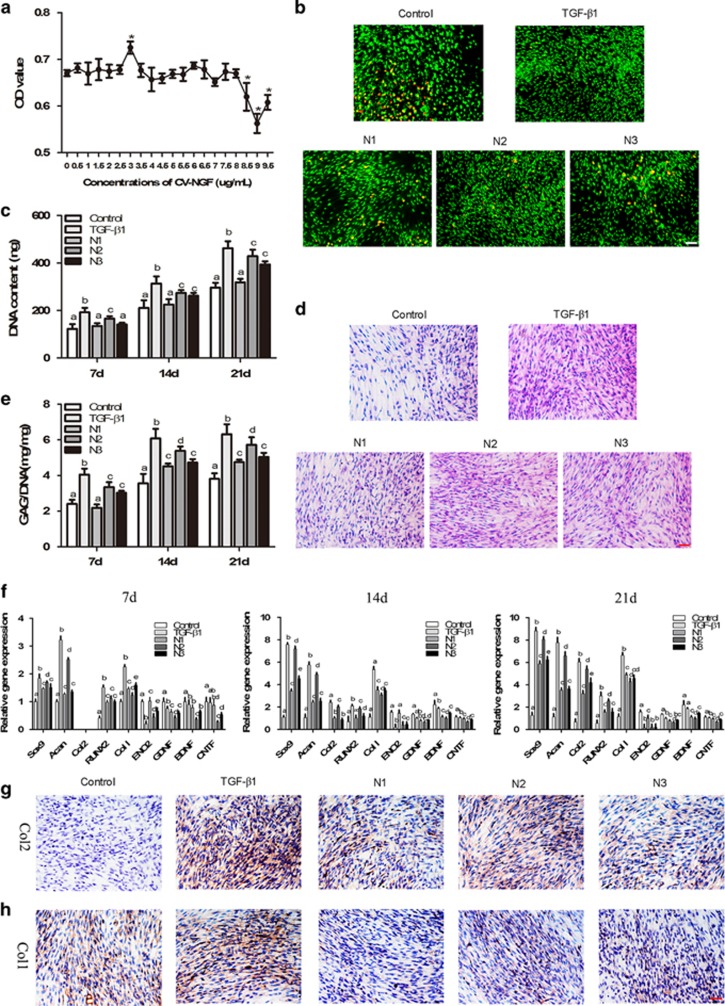
Effects of NGF on BMSCs in monolayer cultures. (**a**) The MTT assay was used to detect the cytotoxic effects of NGF on BMSCs. (The values are means±S.D., *n*=6, *P<0.05). (**b**) The effect of NGF on the viability of BMSCs cultured in monolayers for 21 days. Scale bar, 100 *μ*m. (**c**) Quantification of the DNA content at 7, 14 and 21 days of culture. (**d**) HE staining was used to determine the morphology of BMCSs cultured in monolayers for 21 days. Scale bar, 100 *μ*m. (**e**) GAG secretion from the cells after treatment for 7, 14 and 21 days. (**f**) qRT-PCR was used to determine the expression of the *ACAN*, *SOX9*, *COL2A1*, *COL1A1*, *RUNX2*, *ENO2, GDNF*, *BDNF* and *CNTF* genes on days 7, 14 and 21. (**g** and **h**) Immunohistochemistry. BMSCs were stained with COL1A1 (**g**) and COL2A1 (**h**) antibodies after 21 days in culture. Scale bar, 100 *μ*m. The values are means±S.D., *n*=6; bars with different letters are significantly different from each other at *P<*0.05

*Cell cytotoxicity and cell viability.* We used the MTT assay to evaluate whether NGF affected the growth of BMSCs cultured *in vitro* and to select optimal concentrations that better supported BMSC growth. As shown in Figure 2a, NGF at concentrations of 1.5, 3 and 6 *μ*g/ml, NGF increased BMSC proliferation approximately 4.2%, 8.2% and 4.0%, respectively, compared with the untreated BMSCs. These concentrations were chosen for further investigation.

Live-dead staining was used to determine the effect of NGF on cell viability (Figure 2b). The majority of cells in all groups were stained green, indicating a good viability of cells after 21 days of culture. More live cells and fewer apoptotic cells (shown in red) were present in the NGF groups than in the control group. TGF-*β*1 treatment was comparable with the N2 group, both of which showed improved cell viability compared with the N1 and N3 groups.

#### Cell proliferation and biochemical assay

To study the effect of NGF on BMSC proliferation, the determination of DNA content and HE staining were performed (Figure 2c). The DNA content increased in a time-dependent manner in all of the groups. The NGF-treated groups showed a significantly higher DNA content than the control. Among the NGF-treated groups, the increase in the DNA content was most prominent in the N2 group, with the increases of 35.2%, 30.1% and 44.6% on days 7, 14 and 21, respectively. N2 was similar to TGF-*β*1. This result was also confirmed by HE staining (Figure 2d).

Given the differences in cell number, the GAG content was normalized to DNA content to reveal any differences in the biosynthetic activity of the cells among all groups (Figure 2e). The GAG content was the highest in TGF-*β*1 group at each time point (*P*<0.05). NGF induced a significant increase in GAG accumulation compared with the control except N1 at day 7. Among the NGF-treated groups, N2 elicited more GAG secretion than the other two.

#### Gene expression and secretion of type I and II collagen

The expression of the *ACAN*, *SOX9*, *COL2A1*, *COL1A1*, *RUNX2, ENO2, GDNF*, *BDNF* and *CNTF* was detected by qRT-PCR (Figure 2f). After 14 days, the levels of cartilage-specific genes, including *ACAN*, *SOX9* and *COL2A1*, were notably increased by NGF and TGF-*β*1 compared with the control. In particular, 3 *μ*g/ml NGF induced the highest expression of *ACAN*, *SOX9* and *COL2A1* of all of the NGF groups. *COL1A1* expression was also greatly increased by both NGF and TGF-*β*1. The expression of *COL1A1* in the NGF groups (particularly the N2 group) was significantly lower than that in the TGF-*β*1 group. The expression of *RUNX2*, a key transcription factor associated with hypertrophy and osteoblast differentiation, was similar to that of *COL1A1*. The expression of markers for neural differentiation – *ENO2, GDNF*, *BDNF* and *CNTF*, were not induced by NGF as shown by lower level than the control.

Immunohistochemical staining was used to detect the expression of collagen type I and II after the chondrogenic induction of BMSCs *in vitro*. Large areas of type II collagen-positive staining were observed in the NGF-treated groups, particularly in the N2 group, which approximates the TGF-*β*1 group (Figure 2g). In contrast to type II collagen, type I collagen, which is the marker of fibrocartilage, was more negatively stained in the NGF-treated groups than in TGF-*β*1 group (Figure 2h).

### Chondrogenic effects of NGF on 3D BMSCs cultures

#### Cell viability, cytoskeletal morphology and GAG production

**Figure 3:**
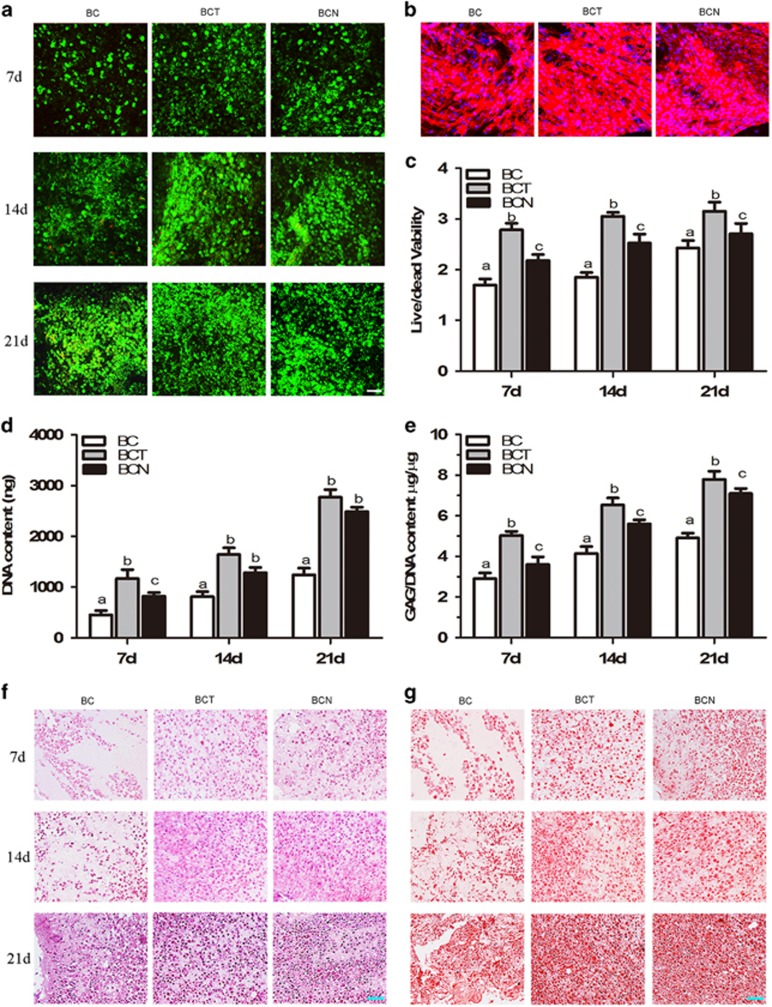
Effects of NGF on BMSCs grown in 3D cultures. (**a**) Cell viability of the cultured 3D constructs on days 7, 14 and 21. (**b**) Phalloidin/Hoechst 33258 stained actin filaments of the constructs on day 21. (**c**) Cell viability was calculated after culture for 7, 14 and 21 days. DNA (**d**) and GAG (**e**) contents of the constructs cultured with different media for 7, 14 and 21 days. (**f** and **g**) HE and Safranin O staining were performed on sections of the cultured constructs on days 7, 14 and 21. The values are means±S.D., *n*=6; bars with different letters are significantly different from each other at *P<*0.05, Scale bar=100 *μ*m

As shown in Figure 3a, live cells (green), which were generally spherical/oval in shape attached to and grew within the hydrogel in a time-dependent manner. More live cells were present in the BCT and BCN groups than control. Comparatively, BCT is better than BCN.

The effect of NGF on cytoskeletal reorganization in 3D cultures of BMSCs at day 21 was investigated by staining with rhodamine–phalloidin and Hoechst 33258. As shown in Figure 3b, a small amount of polymerized actin was distributed in the control cells. In contrast, intensively polymerized actin was observed in both the BCN and BCT groups. Less actin with relatively weaker fluorescence was observed in the BCN group compared with the BCT group.

Biochemical assays were used to quantify the DNA content and GAG production after 7, 14 and 21 days of culture. Both the calculation of live/dead cells (Figure 3c) and the DNA content showed the increased cells with time in all groups (Figure 3d). Compared with those in BC, the number of cells in the BCT and BCN groups increased prominently, with 1.23- and 1.17-fold at day 21, respectively.

As shown in Figure 3e, GAG accumulation in the BCT and BCN groups was significantly increased compared with the BC group (*P*<0.05). Compared with the control, GAG accumulation in the BCN group increased by 9.2%, 45.3% and 53.0% at days 7, 14 and 21, respectively, which was slightly lower than the BCT groups of which increased by 12.3%, 51.7% and 59.0%.

#### Histological and immunohistochemical findings

**Figure 4:**
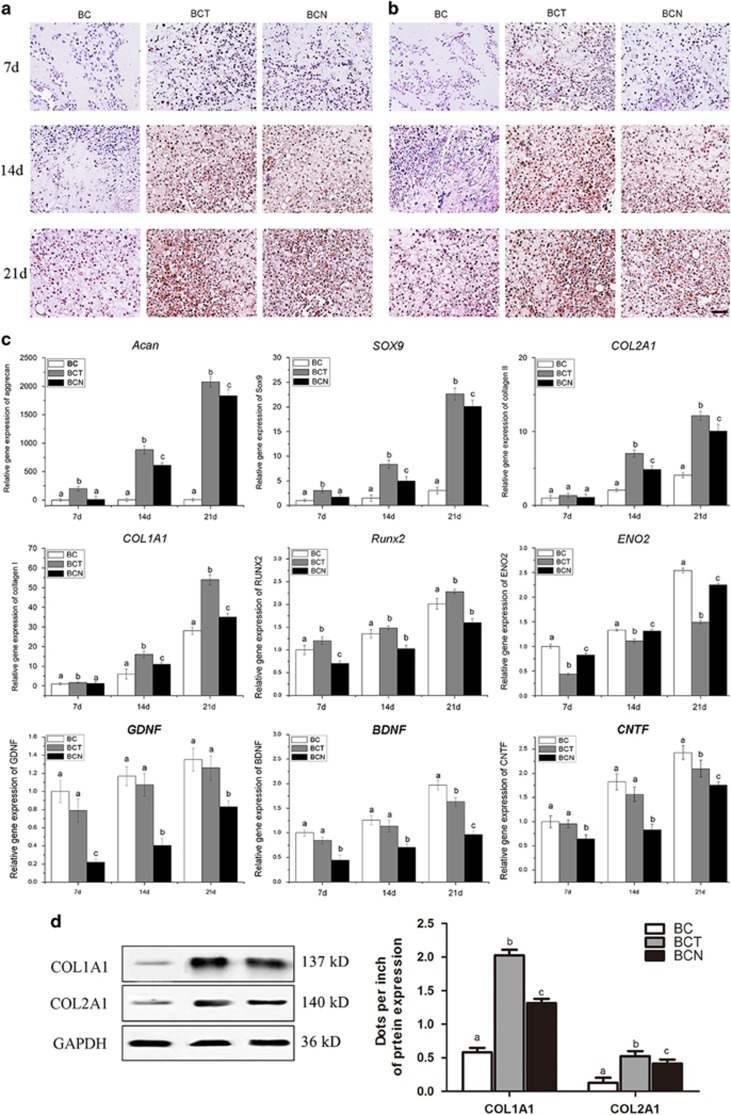
Chondrogenic effects of NGF on BMSCs grown in 3D cultures. (**a** and **b**) Immunohistochemical staining of COL1A1 (**a**) and COL2A1 (**b**) was performed in the cultured constructs on days 7, 14 and 21. (**c**) qRT-PCR was performed to determine the expression levels of *ACAN*, *SOX9*, *COL2A1*, *COL1A1*, *RUNX2, ENO2, GDNF*, *BDNF* and *CNTF* in the cultured constructs on 7 14 and 21 day. (**d**) Western blots was used to analyze the protein expression levels of Col1A1 and COL2A1. The values are means±S.D., *n*=6; bars with different letters are significantly different from each other at *P<*0.05, Scale bar=100 *μ*m

The chondrogenic effects of NGF on BMSCs in 3D culture were evaluated by histological and immunohistochemical staining of cartilage-specific matrices on days 7, 14 and 21. In both the BCN and BCT groups, more cells with the typical features of chondrocytes were embedded in lacuna structures compared with BC group (Figure 3f). Consistent with the GAG content (Figure 3e), safranin O staining indicated that more abundant GAGs were homogeneously distributed in the cells of both the BCN and BCT groups than control (Figure 3g). In addition, stronger positive expression of type II collagen (Figure 4a) and type I collagen (Figure 4b) was observed in the BCN and BCT groups than in the BC group. In comparison, expression of collagen type I and type II in BCT group was abundant than that in BCN groups.

#### qRT-PCR analyses for gene expression in BMSCs seeded on collagen for the induction of chondrogenesis

The chondrogenic differentiation profile of BMSCs grown in 3D cultures was detected by assessing the mRNA expression levels of *ACAN*, *SOX9*, *COL2A1, COL1A1, RUNX2*, *ENO2, GDNF*, *BDNF* and *CNTF* after 7, 14 and 21 days of culture (Figure 4c). The expression of cartilage-specific genes, including *ACAN*, *SOX9* and *COL2A1*, were extensively upregulated in the BCT and BCN groups compared with the BC group. Comparatively, BCT stimulates higher expression of the cartilage-specific genes than BCN. However, lower levels of *COL1A1* and *RUNX2* were observed in the BCN group than in the BCT group. The expression of *ENO2, GDNF*, *BDNF* and *CNTF*, which are markers for neural differentiation, were much lower in both the BCN and BCT groups than in the control. These results were also confirmed by the protein expression levels of collagen I and collagen II (Figure 4d).

### Therapeutic effect of NGF on cartilage defect

#### Gross assessment

**Figure 5:**
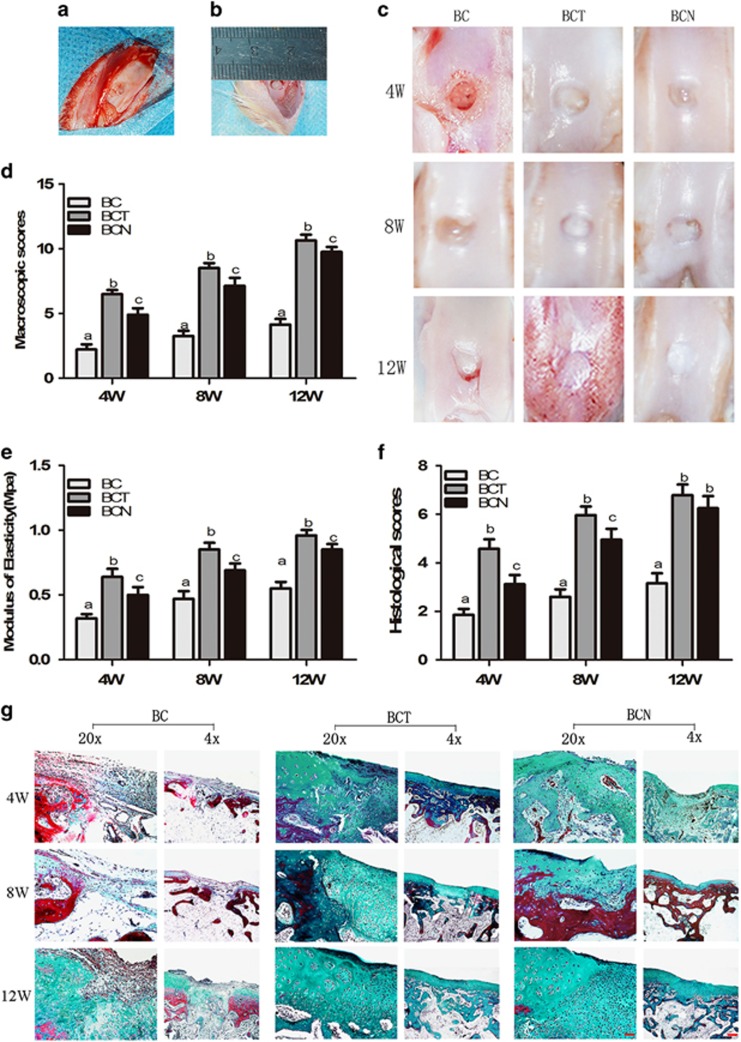
Chondrogenic effects of NGF on BMSCs *in vivo*. (**a** and **b**) Intraoperative photographs showed the location and size of the defect. (**c**) Macroscopic appearance of cartilage defect healing in the three groups at week 12 after surgery. (**d**) International Cartilage Repair Society macroscopic scores for all samples from the three groups at 4, 8 and 12 weeks after surgery. (**e**) Biomechanical testing. (**f**) Histological scores for the samples from the three groups at 4, 8 and 12 weeks after surgery, according to the scale described by Wakitani. (**g**) Masson’s trichrome staining were performed on cartilage sections. The values are means±S.D., *n*=10 joints; bars with different letters are significantly different from each other at *P<*0.05. (Original low magnification × 40 (scale bar, 500 *μ*m); original high magnification × 200 (scale bar, 100 *μ*m))

A cartilage defect model was created by drilling a 4 mm diameter hole in the patellar groove (Figure 5a). Care was taken not to perforate through the cartilaginous layer. Then, the defect was filled with injectable collagen hydrogels seeded with BMSCs (Figure 5b). After 4, 8 and 12 weeks of therapy, the engineered cartilage with part of the subchondral bone was harvested. At 4 weeks of repairing, defects were still grossly distinguishable from the surrounding cartilage tissue in all groups (Figure 5c). In the control, the defect was still evident at 8 weeks and newly formed tissue was not fully filled at 12 weeks. Neo-tissue was formed in the defects after 8 weeks of both the BCN and BCT groups. Twelve weeks post-surgery, glossy and smooth cartilage-like tissues were regenerated and well integrated with the surrounding tissues in both BCT and BCN groups. In order, BCT, BCN and BC exhibited decreased macroscopic scores at each time point (Figure 5d).

#### Biomechanical testing

The compressive stiffness of the repaired tissues from the three groups was determined at 4, 8 and 12 weeks (Figure 5e). The BCN and BCT engineered cartilage was significantly stiffer than the control, with increases of 47.7% and 38.5%, respectively, at week 12. The BC engineered cartilage showed the lowest mechanical strength, indicating the formation of fibrous tissue or fibrocartilage.

#### Histological observation

**Figure 6:**
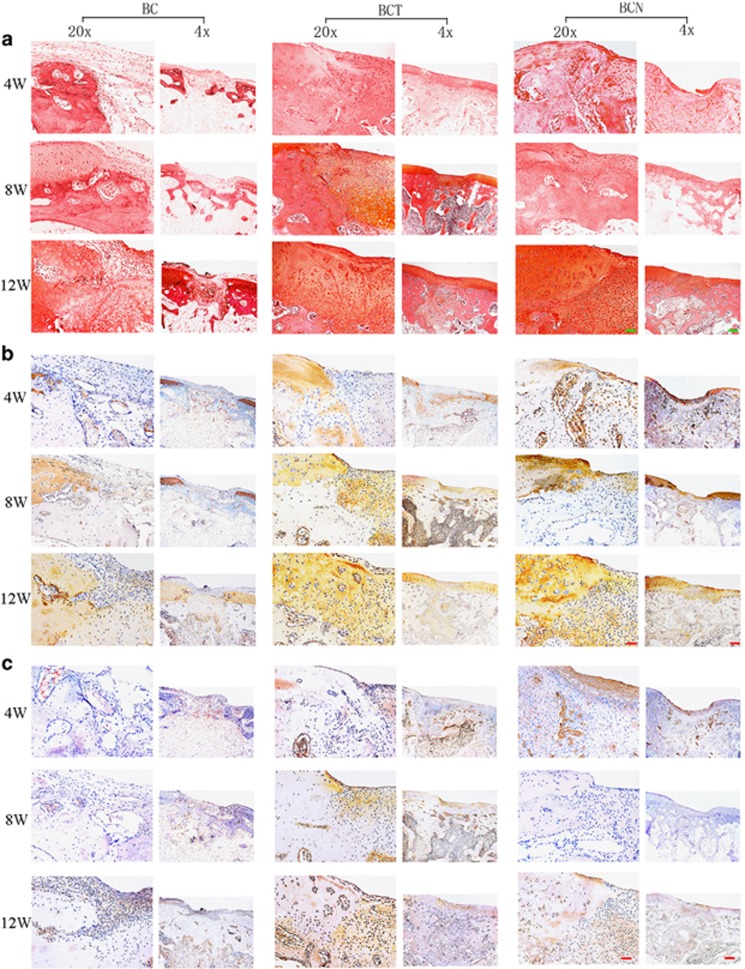
Chondrogenic effects of NGF on BMSCs *in vivo*. (**a**) Safranin O staining were performed on cartilage sections. (**b** and **c**) Immunohistochemical staining of COL1A2 (**b**) and COL1A1 (**c**) were performed in cartilage sections. (Original low magnification × 40 (scale bar, 500 *μ*m); original high magnification × 200 (scale bar, 100 *μ*m))

Upon histological observation after 4, 8 and 12 weeks of therapy, fibrous tissue with a loose and detached interface was observed in the defect of BC group (Figure 5g). In contrast, glossy and smoothly regenerating tissues gradually formed hyaline cartilage-like tissues similar to the surrounding normal cartilage in BCT and BCN groups. The results were also confirmed by the histological scores (Figure 5f). Opposite to the modicum amount in BC groups, the production of GAG increases with time in neocartilages of both BCT and BCN groups, resulting in little difference with surrounding tissue after 12 weeks (Figure 6a). Immunohistochemical staining showed that much more positive staining of collagen type II was present in BCT and BCN groups than control. Comparatively, deeper staining of collagen type I was in BCT than in BCN group.

#### Cartilage-related gene and protein expression

**Figure 7:**
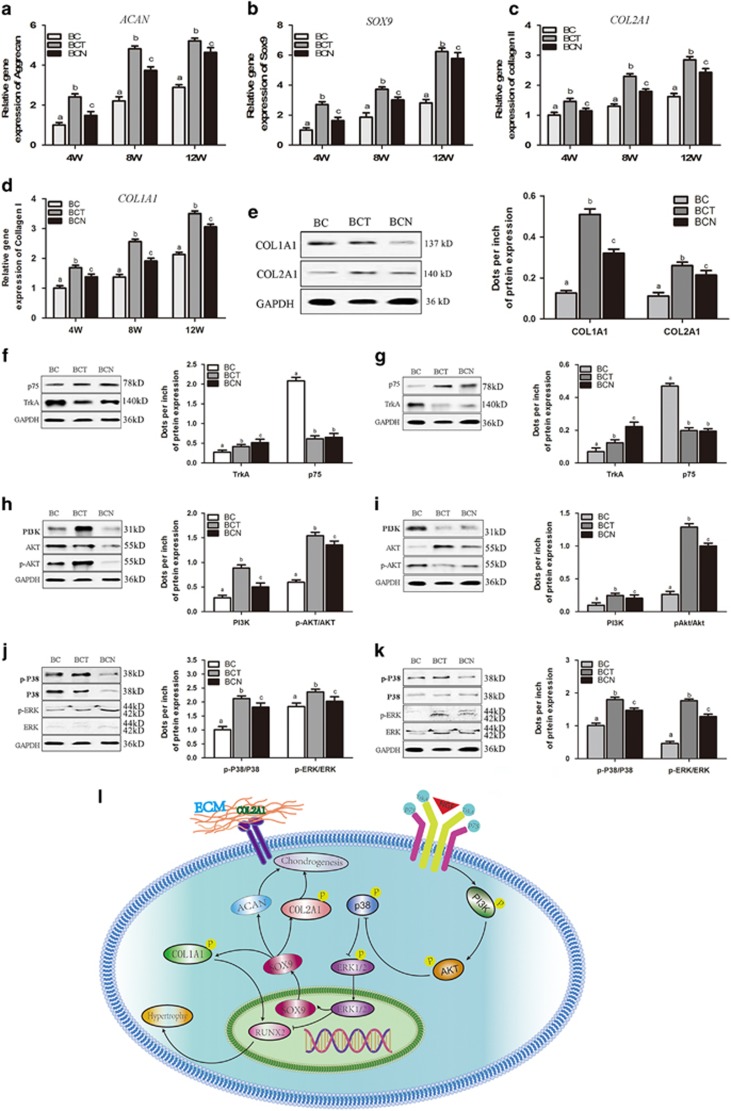
The receptors and signaling pathways that are activated in NGF-induced BMSCs *in vitro* and *in vivo*. (**a-d**) qRT-PCR was to determine the expression levels of the *ACAN*, *SOX9*, *COL2A1* and *COL1A1* genes in the repaired model after 4, 8 and 12 weeks. (**e**) Western blots was used to analyze the protein expression levels of Col1A1 and COL2A1. (**f** and **g**) Western blots was used to analyze the protein expression levels of the NGF receptors TrkA and p75 *in vitro* (**f**) and *in vivo* (**g**). (**h** and **i**) Western blots was used to analyze the protein expression of proteins in the PI3K/AKT signaling pathway, including PI3K, AKT and p-AKT, *in vitro* (**h**) and *in vivo* (**i**). (**j** and **k**) Western blots was used to analyze the expression of proteins in the ERK/MAPK signaling pathway, including ERK, p-ERK, P38 and p-P38, *in vitro* (**j**) and *in vivo* (**k**). (**l)** Schematic description of the relevant signaling pathways that were activated by NGF. The values are means±S.D., *n*=10 joints; bars with different letters are significantly different from each other at *P<*0.05

In agreement with the histological findings, the expression of *ACAN*, *SOX9* and *COL2A1*, were significantly increased in the BCN and BCT engineered cartilage compared with the control (Figures 7a–d). Slightly lower *COL2A1* expression and significantly decreased *COL1A1* expression were observed in BCN compared with BCT. The expression of the collagen I and collagen II proteins also confirmed the qRT-PCR findings (Figure 6b and c).

### Effects of NGF on NGF receptors and chondrogenesis-related signaling pathways

#### Effect of NGF on NGF receptors

TrkA expression was greatly influenced by NGF both *in vitro* (3D culture) (Figure 7f) and *in vivo* (Figure 7g). Both NGF and TGF-*β*1 decreased the expression of p75 ^NTR^ compared with control. There were no significant differences between BCN and BCT in p75 ^NTR^ expression.

#### Chondrogenic effects of NGF on BMSCs are mediated by the PI3K/AKT and MAPK/ERK signaling pathways

To further investigate the regulation of PI3K/AKT or MAPK/ERK signaling pathway in chondrogenesis by NGF, western blot analysis was used to assay the expression of the PI3K, AKT, p-AKT, ERK, p-ERK, P38 and p-P38 proteins. After 21 days of chondrogenic induction in the presence of NGF, the levels of key proteins in both signaling pathways were significantly upregulated compared with the control, although lower than the BCT group (Figures 7h and j). The engineered cartilage in the BCN and BCT groups also exhibited upregulated expression of these proteins (Figures 7i and k).

## Discussion

The yield of NGF in snake venoms is generally approximately 1% or less.^30^ Bian *et al.*^35^ used a two-step method to purify NGF, with the yield of 0.51%. In our previous study, we used two-step method by gel filtration and ion exchange columns to extract NGF.^33, 34^ Although the yield was 0.65%, the NGF was not so pure. In this study, we successfully isolated highly pure NGF from cobra venom with a yield of 0.6% and a purity of 99% by adding a TSK-G2000-SW chromatography step (Figure 1), which can efficiently separate proteins with molecular weights ranging from 5000 to 200 000 Da.^36, 37^ The improved three-step procedure for NGF extraction is easily accessed and is of low cost. Besides, the abundant and inexpensive cobra venom in Southern China greatly decreases the cost. In comparison, recombinant growth factors are much more expensive and complicated to prepare. Thus, highly pure NGF may be easily industrialized by this simplified procedure.

Chondrogenic potential of NGF was confirmed both *in vitro* and *in vivo*, which has not been reported yet. In 2D and 3D cultures, the immunohistochemical and qRT-PCR analyses showed that the expression of cartilage-specific markers, including *ACAN*, *SOX9* and *COL2A1*, was significantly upregulated in the NGF groups compared with the controls (Figures 2 and 4). The 3D cultures also displayed normal features of cartilage, with large numbers of round chondrocytes embedded in the lacuna in the presence of NGF (Figure 3) and the expression of cartilage-specific markers was markedly increased compared with the monolayer culture, which indicated that NGF and 3D scaffold exhibited a synergistic effect. When applied to cartilage repair, NGF also accelerated the healing process, as evidenced by the histological findings, qRT-PCR/WB analyses and biomechanical tests (Figures 5,6, and 7). Tissue engineering technique by only using stem cells and scaffold is useful for the repair of defect. Our study showed that in control groups (BC group), the cartilage defect was gradually repaired with the upregulation of collagen type II over time. However, the healing process is much longer and the therapeutic effect is less satisfiable than that with the assistance of growth factors.^38, 39, 40, 41^Interestingly, NGF was superior to TGF-*β*1 in chondrogenic specificity as evidenced by considerably decreased expression of collagen type I (Figures 2 and 4), a fibrocartilage marker^42^ and *RUNX2* (Figures 2 and 4), a critical for chondrocyte terminal differentiation. This indicates that NGF can better prevent fibrogenic and hypertrophic differentiation to maintain the chondrocytic phenotype of the MSCs and the characteristic of hyaline cartilage may be better retained by NGF. It has been reported that accompanied with chondrogenesis and hypertrophic differentiation,^43^ TGF-*β*1 is also implicated in osteogenic differentiation.^44, 45^ Thus, NGF may be somewhat preferred over TGF-*β*1 regarding its differentiation specificity, although a long-term investigation was needed.

Most studies have shown the potential of NGF to induce the differentiation of stem cells along the neuronal lineage.^46, 47, 48^ However, NGF induced the MSCs to differentiate into the chondrogenic lineage instead of the neuronal linage in this study, as evidenced by no neuronal cells and downregulated expression of neuronal-specific markers^49^ (Figures 2 and 4). This may be a result of the elaborate environment that favors chondrogenesis, such as the high density of cells and the addition of other chondrogenic supplements, such as dexamethasone, ascorbate and ITS. In particular, NGF can greatly increase the expression of cartilage-specific genes and proteins in 3D cultures (Figure 4), which mimic the condensation of mesenchymal cells during chondrogenesis in embryonic development. The results indicated that NGF predominantly induced the stem cells into chondrocytes instead of the neuronal phenotype in an environment favoring chondrogenesis.

The analysis of the molecular mechanisms revealed that several signaling pathways are involved in NGF-induced chondrogenesis, including the MAPK/ERK and PI3K/AKT signaling pathways. The PI3K/AKT signaling pathway has an important role in the physiological effects of NGF^50^ and chondrocyte differentiation.^51, 52^ Both our *in vitro* and *in vivo* results (Figure 7) showed that NGF stimulated PI3K and AKT phosphorylation during the process of MSCs differentiation, resulting in a decrease in the expression of collagen type I and *RUNX2* (Figure 4). The results indicated that the PI3K/AKT signaling pathway was critical for NGF-induced chondrogenesis. Another important signaling pathway, MAPK, P38 MAPK and ERK1/2, are involved in chondrogenic differentiation during adult life.^53, 54, 55^ Here, we report that MAPK activity functionally contributes to NGF-induced chondrogenesis in MSCs, as shown by increased phosphorylation and activity of P38 and ERK1/2 both *in vitro* and *in vivo* (Figure 7). Thus, NGF may induce the chondrogenesis of MSCs by mediation of the MAPK/ERK and PI3K/AKT signaling pathways, which was similar to TGF-*β*1.

TrkA is a specific receptor of NGF and is involved in bone formation and healing.^56^ The binding of NGF stimulates the autophosphorylation and dimerization of TrkA, resulting in the activation of the PI3K pathway and the MAPK pathway.^57^ Signaling via p75^NTR^ is believed to be related to cell apoptosis and growth arrest.^58^ The results of this study showed that TrkA expression in the BCN group was significantly higher than that in the BC group both *in vitro* and *in vivo*, whereas expression of p75^NTR^ was downregulated (Figure 7), which indicated that the chondrogenic effects of NGF on BMSCs may be mediated by the activation of TrkA receptor and the inhibition of p75^NTR^.

Our rationale for introducing NGF into MSC cultures during chondrogenesis for cell-based therapy of cartilage defect was to determine whether we could improve elasticity and minimize dedifferentiation and hypertrophy. The results suggest that NGF triggers the chondrogenic differentiation of MSCs via interactions between NGF and the TrkA/ p75^NTR^ receptors, and these interactions subsequently activate downstream molecules, such as PI3K and AKT. Consequently, the activated PI3K and AKT lead to decreased expression of markers of chondrocyte terminal differentiation (Figure 7). Thus, NGF may be favorable substitute for traditional growth factors in chondrogenesis.

## Materials and Methods

### Preparation of NGF

#### Separation and purity of NGF

Crude cobra venom was sequentially separated on Sephadex G-75, CM Sepharose CL-6B and TSK-G2000-SW chromatography columns. Two grams of crude cobra venom was dissolved in 10 ml of buffer (1% HAc). After centrifugation, the soluble fraction was collected and loaded on the Sephadex G-75 chromatography column. The mobile phase was 1% HAc, with a flow-rate of 2 ml/min. A 10 ml fraction containing NGF activity was obtained and then loaded on the CM Sepharose CL-6B chromatography column after dialyzed overnight. After equilibration with 0.05 M NaAc-HAc (pH=5) buffer, 0.05 M NaCl buffer was used as the mobile phase, with a flow-rate of 1 ml/min. The fraction with NGF activity was collected and then separated by HPLC on the TSK-G2000-SW column. In all, 0.1% (w/v) trifluoroacetic acid (TFA) containing 0.25 M NaCl was used as the mobile phase for linear gradient elution. The fraction with NGF activity was collected, dialyzed for desalination and lyophilized. The UV absorption of the proteins was monitored at 280 nm, with a sample volume of 15 *μl* and a flow-rate (mobile phase) of 1.0 ml/min. NGF was dissolved in normal saline (NS, Sigma-Aldrich, Shanghai, China) at a concentration of 0.1 mg/ml as a stock solution and stored at −20 °C.

#### The molecular weight and identification of NGF

The molecular weight of the chromatography-purified NGF was confirmed by SDS-PAGE. The gel was fixed with 12% trifluoroacetic acid and stained with Coomassie blue R-250.

#### Bioactivity of NGF

Neurite outgrowth from chick embryonic dorsal root ganglia was used to determine the biological activity of NGF. After pre-gummed with rat tail collagen for 3 h at 37°C the dorsal root ganglia were harvested from 8-day-old chick embryos and incubated with 100 ng/ml NGF for 18 h at 37 °C with 5% CO_2_. Root ganglia with no treatment was used as control.

### Animals

A total of 90 female New Zealand white rabbits (weighting 2.5–3 kg and 2 months old) were obtained from Guangxi Medical University, Nanning, China. The rabbits were housed individually at a constant temperature and relative humidity (60%), with free access to a standard diet and water. All experiments were conducted in accordance with the standard guidelines approved by Animal Care and Experiment Committee of Guangxi Medical University (protocol number: 2014-12-3).

### Isolation and culture of BMSCs

BMSCs were harvested from the bone marrow extracted from New Zealand rabbits. The rabbits were anesthetized with 5 mg/ml pentobarbital (30 mg/kg), and a sterile medullo-puncture needle was used to collect the bone marrow from the bilateral femurs. A bone marrow mononuclear cell isolation kit (TBD2013CRA, Tian Jin Hao Yang Biological Manufacture Co., Ltd, Tianjin, China) was used for BMSC extraction. The isolated BMSCs were cultured in alpha-modified Eagle’s medium (Gibco, Thermo Fisher Scientific, Waltham, MA, USA) containing 10% (v/v) fetal bovine serum (Hyclone, Logan, UT, USA) and 1 % (v/v) antibiotics (penicillin 10 000 U/ml and streptomycin 10 000 *μ*g/ml, Solarbio, Beijing, China) under a humidified atmosphere with 5% CO_2_ at 37 °C.

### Scaffold preparation and cell seeding

Collagen type I isolated from calf skin was prepared as described in previous studies.^59, 60^ Collagen type I was dissolved in a CH_3_COOH solution to a final concentration of 10 mg/ml. Then, 0.5 M NaOH was used to neutralize the solution. The cells were detached with 0.25% trypsin/ETDA and centrifuged. BMSCs were loaded in neutralized collagen solution at a final density of 1 × 10^7^ cells/ml. Finally, the cell–matrix constructs were incubated at 37 °C for 10 min to allow gelation.

NGF was added to the cell cultures at various concentrations. For chondrogenic differentiation in 2D cultures, the BMSCs were cultured with chondrogenic medium supplemented with 50 *μ*g/ml ascorbic acid (Sigma), 100 nM dexamethasone (Sigma), a 1% insulin-transferrin-selenium solution (Gibco) and 10 ng/ml TGF-*β*1 (PeproTech, Rocky Hill, PA, USA) (TGF-*β*1 group) or 1.5 *μ*g/ml NGF (N1 group) or 3 *μ*g/ml NGF (N2 group) or 6 *μ*g/ml NGF (N3 group). Cells cultured with no chondrogenic supplements were set as control. For 3D cultures, the BMSCs loaded in collagen constructs were cultured with chondrogenic supplements and 10 ng/ml TGF-*β*1 (BCT group) or NGF of optimal concentration (BCN group) or none (BC group).

### Cytotoxicity assay

The cytotoxic effect of NGF on BMSCs was assessed using an MTT (Gibco) analysis. BMSCs were seeded at a density of 1.56 × 10^4^/cm^2^ in a 96-well plate and cultured with NGF ranging from 1 to 9.5 *μ*g/ml for 24 h. MTT (5 mg/ml) was added to each well and incubated at 37 °C for 4 h. Dimethylsulfoxide (Sigma) was used to dissolve the formed crystals and the absorbance was detected by an Microplate Reader (Thermo Fisher Scientific, Waltham, MA, USA) at 570 nm. All experiments were performed in sextuplicate. As determined by the MTT analysis, optimal concentrations of 1.5, 3 and 6 *μ*g/ml were chosen for further investigation.

### Fluorescence microscopy: viability and F-actin staining

A live/dead cell viability assay kit (Invitrogen Life Technologies, Waltham, MA, USA) was used to evaluate the viability of BMSCs in response to NGF treatment for 7, 14 and 21 days. Cells and cell–matrix constructs were harvested and quickly rinsed with PBS, followed by the incubation with medium containing calceiu-AM and propidium iodide for 5 min in the dark. The images were captured using a laser scanning confocal microscope (Nikon A1, Tokyo, Japan). Live/dead cell viability for the 3D cultured cells was calculated from 2X image using ImageJ software (NIH, Bethesda, MD, USA).

To observe the filamentous actin (F-actin) organization and distribution in the hydrogel, staining for the actin cytoskeleton was performed in all the groups. The cells and BMSC–collagen constructs were washed twice with PBS after 7, 14 and 21 days, and then fixed with 4% paraformaldehyde for 10 min. After washing with PBS, the samples were incubated with 0.1% Triton X-100 for 5 min to permeabilize the cells. Then, the constructs were incubated in rhodamine–phalloidin for 30 min, followed by Hoechst 33258 for 5 min. The images were acquired using a laser scanning confocal microscope (Nikon A1).

### Biochemical assay

After culture for 7, 14 and 21 days, the cells and BMSC–collagen constructs were digested with 60 *μ*g/ml proteinase K (Sigma) for 16 h at 60 °C. To determine the DNA content, the cell lysates were incubated with Hoechst 33258 (Invitrogen, Life Technologies, USA) solution for 5 min. The fluorescence intensity was determined with a spectrofluorometer (Thermo Fisher Scientific, USA) at 460 nm using calf thymus DNA as a standard. To determine the glycosaminoglycan (GAG) content, a colorimetric assay using 1, 9-dimethylmethylene Blue (DMMB; Sigma) dye was performed. The cell lysates by proteinase K were coupled with the DMMB reagent and the absorbance was measured at 525 nm using FlexStation III (Molecular Devices, Sunnyvale, CA, USA). The GAG content was quantified using a standard curve of chondroitin sulfate (Sigma) and normalized to the total DNA content. All experiments were performed in sextuplicate.

### Animal model for the cartilage defect repair studies

Totally 90 New Zealand white rabbits were used for study of cartilage repair. A cartilage-only defect (4 mm diameter) was created in the middle of each patellar groove of the rabbits after general anesthesia. Then, injectable collagen loaded with allogenic BMSCs cultured for 14 days in chondrogenic medium without chondrogenic supplements (BC group, *n*=30) or with 10 ng/ml TGF-*β*1 (BCT group, *n*=30) or 3 *μ*g/ml NGF (BCN group, *n*=30) was injected into the defect of the rabbits. After 4, 8 and 12 weeks of repair, animals were killed and the repaired cartilage samples were harvested for analysis.

### Macroscopic observation

After 4, 8 and 12 weeks of repair, repaired cartilage samples were harvested and photographed. The samples were assessed according to the International Cartilage Repair Society (ICRS) macroscopic assessment scale for cartilage repair.^61^

### Biomechanical test

The compressive strength of the engineered cartilage was analyzed using a compression strength tester (model HY-0230; Shanghai Hengyi Instruments Co., Ltd, Shanghai, China). The repaired articular cartilage was fixed to the apparatus using a metal pin that attached the graft to the tensioner system of the testing machine. Biomechanical loading was assessed after the related parameters were set. The crosshead speed was approximately 0.06 mm/min. The ratio of equilibrium force to cross-sectional area was divided by the applied strain to calculate the equilibrium modulus (in MPa).

### Histological examination

After 7, 14 and 21 days, monolayer cultured cells were fixed with 4% (v/v) paraformaldehyde for 30 min. The 3D cell–gel composites were fixed for 48 h and then embedded in paraffin and cut into 5 *μ*m sections. For the *in vivo* study, after gross inspection, the articular samples were fixed in 4% paraformaldehyde, decalcified, embedded in paraffin and cut into 5 *μ*m sections. The cells and sections were stained with hematoxylin–eosin (HE; JianCheng Biotech, Nanning, China) for a histological evaluation of cell morphology. Safranin O staining was performed to detect GAG accumulation in the 3D constructs and repaired cartilage. Masson’s trichrome staining was used to examine the extent of collagen deposition and fibrosis in the repaired cartilage. An inverted phase contrast microscope (Nikon A1) was used for the histomorphometric and histological observations. The repaired articular cartilage samples were graded using the scale described by Wakitani^62^ by three independent observers (LZ, LD and JT) who were blinded to the conditions to comprehensively evaluate the regeneration of the tissue in the defects.

### Immunohistochemical examination

The secretion of collagen types I and II was detected with an immunohistochemical staining kit (Bioss, Beijking, China). To visualize the proteins, the cells per sections were fixed in 4% (w/v) paraformaldehyde and treated with Triton X-100. The cells per sections were incubated with 3% H_2_O_2_ for 10 min at room temperature to exclude endogenous peroxidase activity. Then, the samples were blocked with normal goat serum for 10 min at room temperature. After 1 : 200 dilutions, mouse anti-rabbit collagen type I (COL1A1, Acris OriGene Technologies, Inc., Rockville, MD, USA, TA342814) and collagen type II (COL2A1, Acris Antibodies GmbH, AF5710) antibodies were added to the cells per ections overnight. Then, the cells per sections incubated with the secondary antibody after washed with PBS. Subsequently, the antibody binding was visualized by a 3, 3’-diaminobenzidine tetrahydrochloride (DAB) kit (Boster, Wuhan, China) before brief counterstaining with hematoxylin. Eventually, the cells per sections were gradually dehydrated, sealed with a neutral gum and observed and photographed with an inverted phase contrast microscope (OLYMPUS Co., Tokyo, Japan).

### RNA extraction and qRT-PCR analysis

Total RNAs of the cells per constructs and cartilage samples were extracted with a Total Isolation RNA kit (Invitrogen) according to the manufacturer’s instructions. The real-time quantitative polymerase chain reaction (qRT-PCR) was used to analyze the expression levels of the aggrecan (*ACAN*), SRY-related high mobility group-box gene 9 (*SOX9*), alpha-1 type II collagen (*COL2A1*), alpha-1 type I collagen (*COL1A1*), enolase 2 (*ENO2*) and runt-related transcription factor 2 (*RUNX2*), enolase 2 (*ENO2*), glial cell-derived neurotrophic factor (GDNF), brain-derived neurotrophic factor (BDNF) and ciliaryneurotrophic factor (CNTF) genes. The primer sequences and GenBank accession numbers used for qRT-PCR are summarized in Table 1. RNA was reverse transcribed into cDNAs using a reverse transcription kit (Fermentas, Hanover, MD, USA). Detection System (RealPlex 4, Eppendorf Corporation, Hamburg, Germany) with a Fast Start Universal SYBR Green Master (Mix, Roche, Basel, Switzerland) at 95 °C for 10 min, followed by 95 °C for 15 s and 60 °C for 1 min. The dissociation curve for each primer pair was analyzed to confirm the primer specificity, and GAPDH was used as an internal control. The expression levels of the target RNAs were calculated based on the threshold cycle (Ct) as R=2^−ΔΔCT^.

### Protein extraction and western blot

Total proteins were extracted with radio immunoprecipitation assay buffer (RIPA, Beyotime, Shanghai, China) containing 1 mM phenylmethylsulfonyl fluoride (Beyotime), according to the manufacturer’s protocols. The proteins were separated by 8% SDS gel electrophoresis and western blotted (*N*=10 joints) using antibodies against NGF (Acris OriGene Technologies, Inc., AM05239PU-N, 1:500), COL1A1 (Acris OriGene Technologies, Inc., TA342814, 1:500), COL2A1 (Bioworld Technology, St. Louis Park, MN, USA, BS1071, 1:800), p75^NTR^ (Acris OriGene Technologies, Inc., TA345845, 1:1000), TrkA (Cell Signaling Technology, Danvers, MA, USA, #2505, 1:800), PI3K (Acris OriGene Technologies, Inc., TA330913, 1:1000), AKT (BIORBYT LTD, Cambridge, UK, ORB235003, 1:500), p-Akt (BIORBYT LTD, 05-802R, 1:800), ERK (Cell Signaling Technology, #4695, 1:800), p-ERK (BIORBYT LTD, T202/Y204, T185/Y187, A303-608A, 1:800), P38 (Acris OriGene Technologies, Inc., TA326350, 1:1000) and p-P38 (BIORBYT LTD, 44-1104G, 1:1000). The blots were incubated with secondary antibodies specific for each protein and quantified using densitometry and standard curves of pure protein.

### Statistical analysis

The data were presented as the means±S.D. and analyzed by one-way analysis of variance (ANOVA) with least significant difference or Dunnett’s *post hoc* multiple comparisons test. Any *P*-values <0.05 were considered to be significant.

## Figures and Tables

**Table 1:** Primer sequences used in qRT-PCR experiments

**mRNA**	**Forward primer**	**Reverse primer**
COL1A1	5′-GTTCAGCTTTGTGGACCTCCG-3′	5′-GCAGTTCTTGGTCTCGTCAC-3′
COL2A1	5′-AAGCTGGTGAGAAGGGACTG-3′	5′-GGAAACCTCGTTCACCCCTG-3′
ACAN	5′-CTACACGCTACACCCTCGAC-3′	5′-ACGTCCTCACACCAGGAAAC-3′
SOX9	5′-AAGCTCTGGAGACTTCTGAACG-3′	5′-CGTTCTTCACCGACTTCCTCC-3′
RUNX2	5′-TCAGGCATGTCCCTCGGTAT-3′	5′-TGGCAGGTAGGTATGGTAGTGG-3′
ENO2	5′-TGTGGTCATGCCTGCCTAAG -3′	5′-GCCACGTGAAATACAGACGC-3′
GDNF	5′-TGTGGTCATGCCTGCCTAAG-3′	5′-GCCACGTGAAATACAGACGC-3′
BDNF	5′-AAGCAAACGTCCGAGGACAA-3′	5′-GAGGCTCCAAAGGCACTTGA-3′
CNTF	5′-CAGACCTGACCGCTCTTACG-3′	5′-GGAGGTTCTCTTGGAGTCGC-3′

## References

[bib1] Caplan AI. Adult mesenchymal stem cells for tissue engineering versus regenerative medicine. J Cell Physiol 2007; 213: 341–347. 17620285 10.1002/jcp.21200

[bib2] Yeh T, Wu S, Lee C, Wen Z, Lee H, Yang Z et al. The short-term therapeutic effect of recombinant human bone morphogenetic protein-2 on collagenase-induced lumbar facet joint osteoarthritis in rats. Osteoarthritis Cartilage 2007; 15: 1357. 17590359 10.1016/j.joca.2007.04.019

[bib3] van Beuningen HM, Glansbeek HL, van der Kraan PM, van den Berg WB. Differential effects of local application of BMP-2 or TGF-β1 on both articular cartilage composition and osteophyte formation. Osteoarthritis Cartilage 1998; 6: 306–317. 10197165 10.1053/joca.1998.0129

[bib4] Blaney Davidson EN, Vitters EL, van Beuningen HM, van de Loo FA, van den Berg WB, van der Kraan PM. Resemblance of osteophytes in experimental osteoarthritis to transforming growth factor beta-induced osteophytes: limited role of bone morphogenetic protein in early osteoarthritic osteophyte formation. Arthritis Rheum 2007; 56: 4065–4073. 18050218 10.1002/art.23034

[bib5] Bakker AC, van de Loo FA, van Beuningen HM, Sime P, van Lent PL, van der Kraan PM et al. Overexpression of active TGF-beta-1 in the murine knee joint: evidence for synovial-layer-dependent chondro-osteophyte formation. Osteoarthritis Cartilage 2001; 9: 128–136. 11237660 10.1053/joca.2000.0368

[bib6] van Beuningen HM, Glansbeek HL, van der Kraan PM, van den Berg WB. Osteoarthritis-like changes in the murine knee joint resulting from intra-articular transforming growth factor-beta injections. Osteoarthritis Cartilage 2000; 8: 25–33. 10607496 10.1053/joca.1999.0267

[bib7] Grassel S. The role of peripheral nerve fibers and their neurotransmitters in cartilage and bone physiology and pathophysiology. Arthritis Res Ther 2014; 16: 485. 25789373 10.1186/s13075-014-0485-1 PMC4395972

[bib8] Kido MA, Kiyoshima T, Kondo T, Ayasaka N, Moroi R, Terada Y et al. Distribution of substance P and calcitonin gene-related peptide-like immunoreactive nerve fibers in the rat temporomandibular joint. J Dental Res 1993; 72: 592–598. 10.1177/00220345930720030701 7680675

[bib9] Letic-Gavrilovic A, Piattelli A, Abe K. Nerve growth factor beta(NGF beta) delivery via a collagen/hydroxyapatite (Col/HAp) composite and its effects on new bone ingrowth. J Materials Sci Materials Med 2003; 14: 95–102. 10.1023/a:1022099208535 15348479

[bib10] Mehrholz J, Rutte K, Pohl M. Jump training is feasible for nearly ambulatory patients after stroke. Clin Rehabil 2006; 20: 406–412. 16774091 10.1191/0269215506cr954oa

[bib11] Lee M, Siu RK, Ting K, Wu BM. Effect of Nell-1 delivery on chondrocyte proliferation and cartilaginous extracellular matrix deposition. Tissue Engineering Part A 2010; 16: 1791–1800. 20028218 10.1089/ten.TEA.2009.0384

[bib12] Xiao D, Hu J, Chen K, Man C, Zhu SS. Protection of articular cartilage by intra-articular injection of NEL-like molecule 1 in temporomandibular joint osteoarthritis. J Craniofac Surg 2012; 23: E55–E58. 22337466 10.1097/SCS.0b013e3182418d02

[bib13] Chen W, Zhang X, Siu RK, Chen F, Shen J, Zara JN et al. Nfatc2 is a primary response gene of nell‐1 regulating chondrogenesis in ATDC5 cells. J Bone Mineral Res 2011; 26: 1230–1241. 10.1002/jbmr.314 PMC3312756 21611965

[bib14] Kawamoto K, Matsuda H. Nerve growth factor and wound healing. Progr Brain Res 2004; 146: 369. 10.1016/S0079-6123(03)46023-8 14699974

[bib15] Cantarella G, Lempereur L, Presta M, Ribatti D, Lombardo G, Lazarovici P et al. Nerve growth factor–endothelial cell interaction leads to angiogenesis *in vitro* and *in vivo*. FASEB J 2002; 16: 1307–1309. 12154004 10.1096/fj.01-1000fje

[bib16] Blanco-Mezquita T, Martinez-Garcia C, Proenca R, Zieske JD, Bonini S, Lambiase A et al. Nerve growth factor promotes corneal epithelial migration by enhancing expression of matrix metalloprotease-9. Invest Ophthalmol Visual Sci 2013; 54: 3880–3890. 23640040 10.1167/iovs.12-10816 PMC5110072

[bib17] Vera C, Tapia V, Kohan K, Gabler F, Ferreira A, Selman A et al. Nerve growth factor induces the expression of chaperone protein calreticulin in human epithelial ovarian cells. Hormone Metabolic Res 2012; 44: 639–643. 10.1055/s-0032-1311633 22773372

[bib18] Micera A, Lambiase A, Stampachiacchiere B, Sgrulletta R, Normando EM, Bonini S et al. Nerve growth factor has a modulatory role on human primary fibroblast cultures derived from vernal keratoconjunctivitis-affected conjunctiva. Mol Vision 2007; 13: 981. PMC2774460 17653039

[bib19] Tuszynski MH, Peterson DA, Ray J, Baird A, Nakahara Y, Gage FH. Fibroblasts genetically modified to produce nerve growth factor induce robust neuritic ingrowth after grafting to the spinal cord. Exp Neurol 1994; 126: 1. 8157119 10.1006/exnr.1994.1037

[bib20] Kawamura M, Urist MR. Growth factors, mitogens, cytokines, and bone morphogenetic protein in induced chondrogenesis in tissue culture. Dev Biol 1988; 130: 435–442. 3058540 10.1016/0012-1606(88)90339-9

[bib21] Gigante A, Bevilacqua C, Pagnotta A, Manzotti S, Toesca A, Greco F. Expression of NGF, Trka and p75 in human cartilage. Eur J Histochem 2009; 47: 339–344. 14706929

[bib22] Iannone F, De Bari C, Dell'Accio F, Covelli M, Patella V, Bianco GL et al. Increased expression of nerve growth factor (NGF) and high affinity NGF receptor (p140 TrkA) in human osteoarthritic chondrocytes. Rheumatology 2002; 41: 1413–1418. 12468822 10.1093/rheumatology/41.12.1413

[bib23] Grimsholm O, Guo Y, Ny T, Forsgren S. Expression patterns of neurotrophins and neurotrophin receptors in articular chondrocytes and inflammatory infiltrates in knee joint arthritis. Cells Tissues Organs 2008; 188: 299–309. 18349525 10.1159/000121432

[bib24] Chao MV, Rajagopal R, Lee FS. Neurotrophin signalling in health and disease. Clin Sci 2006; 110: 167–173. 16411893 10.1042/CS20050163

[bib25] Reichardt LF. Neurotrophin-regulated signalling pathways. Philos Trans R Soc Lond B Biol Sci 2006; 361: 1545–1564. 16939974 10.1098/rstb.2006.1894 PMC1664664

[bib26] Aloe L, Rocco ML, Bianchi P, Manni L. Nerve growth factor: from the early discoveries to the potential clinical use. J Transl Med 2012; 10: 1–15. 23190582 10.1186/1479-5876-10-239 PMC3543237

[bib27] Lee H-H, Chang C-C, Shieh M-J, Wang J-P, Chen Y-T, Young T-H et al. Hypoxia enhances chondrogenesis and prevents terminal differentiation through PI3K/Akt/FoxO dependent anti-apoptotic effect. Sci Rep-Uk 2013; 3: 2683. 10.1038/srep02683 PMC3775095 24042188

[bib28] Ciarmatori S, Kiepe D, Haarmann A, Huegel U, Tönshoff B. Signaling mechanisms leading to regulation of proliferation and differentiation of the mesenchymal chondrogenic cell line RCJ3. 1C5. 18 in response to IGF-I. J Mol Endocrinol 2007; 38: 493–508. 17446238 10.1677/jme.1.02179

[bib29] Kilmon J. Snake venom:" gentler" purification provides attractive nerve growth factor source. Am Biotechnol Lab 1992; 10: 18 20. 1368851

[bib30] Paalme V, Trummal K, Samel M, Tõnismägi K, Järvekülg L, Vija H et al. Nerve growth factor from< i> Vipera lebetina</i> venom. Toxicon 2009; 54: 329–336. 19463841 10.1016/j.toxicon.2009.05.001

[bib31] Tong Q, Wang F, Zhou H-Z, Sun H-L, Song H, Shu Y-Y et al. Structural and functional insights into lipid-bound nerve growth factors. FASEB J 2012; 26: 3811–3821. 22649032 10.1096/fj.12-207316

[bib32] Chen L, Wang Y, Deng G, Yang L. Effect of NGF, which is isolated and purified from the venom of Naja naja atra, on expression of GAP-43 in dorsal root ganglia removed partially from cat. Sichuan da xue xue bao Yi xue ban J Sichuan Univ Med Sci Ed 2007; 38: 421–423 455. 17593821

[bib33] Li XB, Chen MJ, Lei DQ, Yang B, Liao GS, Shu YY et al. Bioactivities of nerve growth factor from Chinese cobra venom. J Natural Toxins 1999; 8: 359–362. 10591039

[bib34] Li XB, Liao GS, Shu YY, Tang SX. Brain delivery of biotinylated NGF bounded to an avidin-transferrin conjugate. J Natural Toxins 2000; 9: 73–83. 10701183

[bib35] Bian L-j, Wu P, Yang X-y. Two-step chromatographic method for separation and purification of nerve growth factor from venom of Chinese cobra. J Chromatogr B 2004; 805: 119–125. 10.1016/j.jchromb.2004.02.030 15113547

[bib36] Regnier FE, Gooding KM. High-performance liquid chromatography of proteins. Anal Biochem 1980; 103: 1–25. 6990825 10.1007/978-1-4615-6728-8_1

[bib37] Irvine GB, Shaw C. High-performance gel permeation chromatography of proteins and peptides on columns of TSK-G2000-SW and TSK-G3000-SW: a volatile solvent giving separation based on charge and size of polypeptides. Anal Biochem 1986; 155: 141–148. 3013045 10.1016/0003-2697(86)90239-3

[bib38] Chen P, Tao J, Zhu S, Cai Y, Mao Q, Yu D et al. Radially oriented collagen scaffold with SDF-1 promotes osteochondral repair by facilitating cell homing. Biomaterials 2015; 39: 114–123. 25477178 10.1016/j.biomaterials.2014.10.049

[bib39] Zhang W, Chen J, Tao J, Jiang Y, Hu C, Huang L et al. The use of type 1 collagen scaffold containing stromal cell-derived factor-1 to create a matrix environment conducive to partial-thickness cartilage defects repair. Biomaterials 2013; 34: 713–723. 23107295 10.1016/j.biomaterials.2012.10.027

[bib40] Wang W, Sun L, Zhang P, Song J, Liu W. An anti-inflammatory cell-free collagen/resveratrol scaffold for repairing osteochondral defects in rabbits. Acta Biomater 2014; 10: 4983–4995. 25169257 10.1016/j.actbio.2014.08.022

[bib41] Zheng L, Fan HS, Sun J, Chen XN, Wang G, Zhang L et al. Chondrogenic differentiation of mesenchymal stem cells induced by collagen-based hydrogel: an *in vivo* study. J Biomed Materials Res Pt A 2010; 93: 783–792. 10.1002/jbm.a.32588 19653302

[bib42] Smeriglio P, Dhulipala L, Lai JH, Goodman SB, Dragoo JL, Smith RL et al. Collagen VI enhances cartilage tissue generation by stimulating chondrocyte proliferation. Tissue Engineering Pt A 2014; 21: 840–849. 10.1089/ten.TEA.2014.0375 25257043

[bib43] Lee CSD, Watkins E, Burnsed OA, Schwartz Z, Boyan BD. Tailoring adipose stem cell trophic factor production with differentiation medium components to regenerate chondral defects. Tissue Engineering Pt A 2013; 19: 1451–1464. 10.1089/ten.tea.2012.0233 PMC3638517 23350662

[bib44] Mastrogiacomo M, Cancedda R, Quarto R. Effect of different growth factors on the chondrogenic potential of human bone marrow stromal cells. Osteoarthritis Cartilage 2001; 9: S36–S40. 11680686 10.1053/joca.2001.0442

[bib45] Heng BC, Cao T, Lee EH. Directing stem cell differentiation into the chondrogenic lineage *in vitro*. Stem Cells 2004; 22: 1152–1167. 15579636 10.1634/stemcells.2004-0062

[bib46] Colafrancesco V, Villoslada P, Targeting NGF. pathway for developing neuroprotective therapies for multiple sclerosis and other neurological diseases. Arch Italiennes de Biologie 2011; 149: 183–192. 10.4449/aib.v149i2.1376 21701990

[bib47] Ding J, Cheng Y, Gao S, Chen J. Effects of nerve growth factor and Noggin-modified bone marrow stromal cells on stroke in rats. J Neurosci Res 2011; 89: 222–230. 21162129 10.1002/jnr.22535

[bib48] Yuan J, Huang GR, Xiao Z, Lin LB, Han TW. Overexpression of beta-NGF promotes differentiation of bone marrow mesenchymal stem cells into neurons through regulation of AKT and MAPK pathway. Mol Cell Biochem 2013; 383: 201–211. 23934089 10.1007/s11010-013-1768-6

[bib49] Zhao HB, Ma H, Ha XQ, Zheng P, Li XY, Zhang M et al. Salidroside induces rat mesenchymal stem cells to differentiate into dopaminergic neurons. Cell Biol Int 2014; 38: 462–471. 24323403 10.1002/cbin.10217 PMC4410750

[bib50] Wang WX, Hu XY, Xie XJ, Liu XB, Wu RR, Wang YP et al. Nerve growth factor induces cord formation of mesenchymal stem cell by promoting proliferation and activating the PI3K/Akt signaling pathway. Acta Pharmacol Sin 2011; 32: 1483–1490. 22139028 10.1038/aps.2011.141 PMC4010210

[bib51] Lee HH, Chang CC, Shieh MJ, Wang JP, Chen YT, Young TH et al. Hypoxia enhances chondrogenesis and prevents terminal differentiation through PI3K/Akt/FoxO dependent anti-apoptotic effect. Sci Rep-Uk 2013; 3: 2683. 10.1038/srep02683 PMC3775095 24042188

[bib52] Kita K, Kimura T, Nakamura N, Yoshikawa H, Nakano T. PI3K/Akt signaling as a key regulatory pathway for chondrocyte terminal differentiation. Genes Cells 2008; 13: 839–850. 18782222 10.1111/j.1365-2443.2008.01209.x

[bib53] Zhao Y-H, Lv X, Liu Y-L, Zhao Y, Li Q, Chen Y-J et al. Hydrostatic pressure promotes the proliferation and osteogenic/chondrogenic differentiation of mesenchymal stem cells: the roles of RhoA and Rac1. Stem Cell Res 2015; 14: 283–296. 25794483 10.1016/j.scr.2015.02.006

[bib54] Zhang Y, Pizzute T, Pei M. A review of crosstalk between MAPK and Wnt signals and its impact on cartilage regeneration. Cell Tissue Res 2014; 358: 633–649. 25312291 10.1007/s00441-014-2010-x PMC4234693

[bib55] McMahon LA, Prendergast PJ, Campbell VA. A comparison of the involvement of p38, ERK1/2 and PI3K in growth factor-induced chondrogenic differentiation of mesenchymal stem cells. Biochem Biophys Res Commun 2008; 368: 990–995. 18267113 10.1016/j.bbrc.2008.01.160

[bib56] Rapp AE, Kroner J, Baur S, Schmid F, Walmsley A, Mottl H et al. Analgesia via blockade of NGF/TrkA signaling does not influence fracture healing in mice. J Orthop Res 2015; 33: 1235–1241. 25876530 10.1002/jor.22892

[bib57] Ehlers MD, Kaplan DR, Price DL, Koliatsos VE. NGF-stimulated retrograde transport of trkA in the mammalian nervous system. J Cell Biol 1995; 130: 149–156. 7540615 10.1083/jcb.130.1.149 PMC2120503

[bib58] Hamanoue M, Middleton G, Wyatt S, Jaffray E, Hay RT, Davies AM. p75-mediated NF-κB activation enhances the survival response of developing sensory neurons to nerve growth factor. Mol Cell Neurosci 1999; 14: 28–40. 10433815 10.1006/mcne.1999.0770

[bib59] Zheng L, Sun J, Chen X, Wang G, Jiang B, Fan H et al. *In vivo* cartilage engineering with collagen hydrogel and allogenous chondrocytes after diffusion chamber implantation in immunocompetent host. Tissue Engineering Pt A 2009; 15: 2145–2153. 10.1089/ten.tea.2008.0268 19326967

[bib60] Zheng L, Fan H, Sun J, Chen X, Wang G, Zhang L et al. Chondrogenic differentiation of mesenchymal stem cells induced by collagen‐based hydrogel: an *in vivo* study. J Biomed Materials Res Pt A 2010; 93: 783–792. 10.1002/jbm.a.32588 19653302

[bib61] Jiang Y, Chen LK, Zhu DC, Zhang GR, Guo C, Qi YY et al. The inductive effect of bone morphogenetic protein-4 on chondral-lineage differentiation and in situ cartilage repair. Tissue Engineering Pt A 2010; 16: 1621–1632. 10.1089/ten.TEA.2009.0681 20001220

[bib62] Wakitani S, Goto T, Pineda SJ, Young RG, Mansour JM, Caplan AI et al. Mesenchymal cell-based repair of large, full-thickness defects of articulsar cartilage. J Bone Joint Surg Am 1994; 76: 579–592. 8150826 10.2106/00004623-199404000-00013

